# 881. Gender in Burn Care: Different Injuries, Equal Outcomes

**DOI:** 10.1093/jbcr/irag033.367

**Published:** 2026-04-06

**Authors:** Anjay Khandelwal, Kaitlyn Malek, Tony Zhao

**Affiliations:** Akron Children's Hospital, Akron, OH; Northeast Ohio Medical University, Akron, OH; Northeast Ohio Medical University, Akron, OH

## Abstract

**Introduction:**

Globally, burn injuries remain a major source of mortality and disability. Prior studies suggest women experience a higher incidence of burns and face barriers to equitable care, raising concern for outcome disparities. To evaluate whether such disparities exist within a modern U.S. regional burn center, we conducted a retrospective outcomes analysis.

**Methods:**

A retrospective review of all burn inpatients (2022–2023) was performed. Collected variables included demographics (age, sex, race), %TBSA, burn etiology, and outcomes (number of surgical procedures, ventilator days, LOS, and actual hospital costs). Propensity score matching (age, TBSA, etiology) was used to balance groups. Associations of gender with outcomes were analyzed using univariate and multivariable quantile regression.

**Results:**

The cohort included 465 patients (157 females, 308 males). Burn etiology differed by sex: scalds were most common in females (48.4%), while flame burns predominated in males (45.5%). Females were significantly younger than males (33.5 vs. 39.3 years, *p*=.02). Females had smaller TBSA burns, shorter LOS, and fewer procedures, though these differences were not statistically significant. Average actual hospital costs were significantly lower in females ($49 316) compared to males ($65 588, *p*=.02).

Univariate analyses showed sex was not a significant predictor of LOS, procedures, ventilator days, or cost. Multivariable models confirmed that TBSA and flame burns, not sex, were the strongest predictors of longer LOS, higher ventilator use, and greater costs.

**Conclusions:**

While prior literature highlights gender-based disparities in burn incidence and care access, our study found no significant sex-related differences in clinical outcomes once patients reached specialized care. Instead, burn size and flame injuries remained the dominant drivers of cost and resource use. Importantly, gender differences in burn etiology—scalds among women and flame burns among men—highlight distinct risk profiles requiring tailored prevention strategies.

**Applicability of Research to Practice:**

These findings reinforce that equitable outcomes are achievable in specialized burn care. However, prevention efforts must remain gender-sensitive, addressing the specific epidemiologic risks faced by men and women. Burn centers should continue to monitor outcomes across demographic groups to ensure equity and proactively identify emerging disparities.

**Funding for the study:**

N/A.

